# Time-lapse 3D imaging by positron emission tomography of Cu mobilized in a soil column by the herbicide MCPA

**DOI:** 10.1038/s41598-018-25413-9

**Published:** 2018-05-04

**Authors:** Johannes Kulenkampff, Madeleine Stoll, Marion Gründig, Alexander Mansel, Johanna Lippmann-Pipke, Michael Kersten

**Affiliations:** 1 Helmholtz Zentrum Dresden-Rossendorf (HZDR), Institute of Resource Ecology, Research Site Leipzig, Permoserstrasse 15, 04318 Leipzig, Germany; 2 Geosciences Institute, Johannes Gutenberg University, J.-J. Becherweg 21, 55099 Mainz, Germany; 3 Helmholtz-Institute Freiberg for Resource Technology, Metallurgy and Recycling Division, Chemnitzer Str. 40, 09599 Freiberg, Germany; 4 Present Address: Institute of Nuclear Waste Disposal, Karlsruhe Institute of Technology, Hermann-von-Helmholtz-Platz 1, 76344 Eggenstein-Leopoldshafen, Germany; 5 Present Address: Federal Institute for Geosciences and Natural Resources (BGR), Rock Characterization for Storage and Final Disposal, Stilleweg 2, 30655 Hannover, Germany

## Abstract

Phenoxyalkanoic acids like the 4-chloro-2-methylphenoxyacetic acid (MCPA) are the second highest used xenobiotic herbicides worldwide after glyphosate because of their apparently favorable environmental properties. Experimental batch equilibration data suggested a reduced Cu adsorption efficiency with the soil mineral goethite below pH 6 in presence of MCPA. This has been verified by advanced surface complexation adsorption modelling involving dissolved Cu-MCPA complexation constants. Positron emission tomography is a non-invasive molecular imaging method for time-resolved three-dimensional information commonly applied on non-retarded tracers in soil core scale experiments. Mineral surface reactive tracers like Cu-64 are too immobile for the relatively short observation times available with this advanced imaging technique. However, Cu-64 radiolabeled Cu-MCPA complex migration could be observed in as long as 10-cm artificial soil test columns where break-through occurred within a few days. For the first time, time-lapse movies of Cu migration in the opaque soil columns were recorded using this novel reactive transport process tomography approach.

## Introduction

Agrochemical formulations based on phenoxyalkanoic acids are used as herbicides to control the spread of annual and perennial broadleaved weeds in crops (including cereals) and lawns. This herbicide class act as synthetic antagonists of auxins^1^ to prevent unwanted weed growth. They are the second most widely applied herbicide (after glyphosate) worldwide. The dosage in European Union countries is usually on the order of 1 kg/ha. There is some debate about their toxicological properties, since commercial formulations may involve adjuvants, safeners, and other utility modifiers, some of which may cause non-target toxic effects other than the pure active substance alone^2^. The latter has definitely favorable environmental properties, because it has a polar functional group and a relatively low environmental persistence allowing “smarter pest control”^3^. The known degradation and adsorption parameters allows estimation of the moderate leaching potential using the Groundwater Ubiquity Score (GUS), which for MCPA lies between 1.6 and 2.9^4^. On the other hand, as shown in one of few alerting studies, MCPA application to acidic (KCl-pH 5.0) sandy loam soil significantly increased the Cu concentration in the roots of wheat plants^2^. Increased soil Cu concentrations are caused by the application of Cu-containing fertilizers^5^ to soils deficient in available micronutrients, the application of sewage sludge, and particularly the application of large amounts of Cu-containing inorganic fungicides (e.g., the Bordeaux mixture that has been used to control mildew in vineyards for 200 years). The fate of Cu in soil is strongly affected by soil solution parameters such as the pH and complexing dissolved components like fulvic acids. Our hypothesis was that organic herbicides like MCPA can increase the dissolved Cu concentration in soil porewater by complexing the Cu as well. They may outcompete the otherwise efficient retardation of Cu by adsorption onto typical soil minerals like ferric oxyhydroxides. This issue will first be explored in more detail in this paper using advanced surface complexation modelling.

Dynamic *in-situ* imaging techniques^6^ are key for better understanding of solute component migration behavior in soil and aquifer rock material. Radioisotopes are commonly used as probes for such reactive process imaging. Nuclear medical imaging tools like gamma camera, single photon or positron emission tomography (SPECT, PET) enable to capture the full three-dimensional complexity and dynamics of solute transport in the porous structure of geomaterials. These tomographic imaging techniques have already been used successfully in previous studies^7–15^ to extract effective fluid flow parameters. This was done by aligning numerical (but not reactive) transport model results to tracer imaging data. However, these studies were limited to radioisotopes of non-reactive elements (i.e., not expected to be retarded by adsorption onto mineral surfaces, like ^18^F, ^22^Na, ^124^I, and ^99m^Tc(VII)). Our molecular imaging approach is novel in that we used it to study the migration of the chemically reacting (i.e., particle surface reactive) trace metal Cu. The greatest obstacle for visualizing three-dimensional reactive tracer flows by means of SPECT or PET is the relatively short decay times of the radioisotope tracers (e.g., T_1/2_ = 12.7 h for ^64^Cu). A drastic acceleration of the reactive tracer migration in a soil column to the order of a couple cm per day is therefore necessary to apply successfully radiotracer imaging. The acceleration found in this study was complexation of Cu by the common herbicide 4-chloro-2-methylphenoxyacetic acid (MCPA).

## Results

### Adsorption Experiments and Modeling

Reviews^4,16^ of over 100 published distribution coefficients has indicated a relatively low mean log*K*_d_ value of −0.10 ± 0.56 L kg^−1^ for MCPA. Significant correlation between the *K*_d_ values and the contents of Fe hydroxides has been reported in these studies. Better model predictions for pH-dependent adsorption were obtained when Fe-bearing mineral soil components were included^17,18^. Common ferric oxy-hydroxides in soil like ferrihydrite or goethite are often coated with humic matter, but this has been found^19^ not to markedly increase their sorption capacities for MCPA at ambient pH values. Kersten *et al*.^20^ performed a molecular modeling study and found that hydrophilic sorption of MCPA by goethite can be explained through strong inner-sphere surface complexation at low pH values, and relatively weak outer-sphere surface complexation at pH > 4. With this background information at hand, pH-dependent batch equilibrium adsorption experiments have been performed with Cu incorporating MCPA and the Fe oxy-hydroxide goethite used in the artificial soil columns.

**Figure 1:**
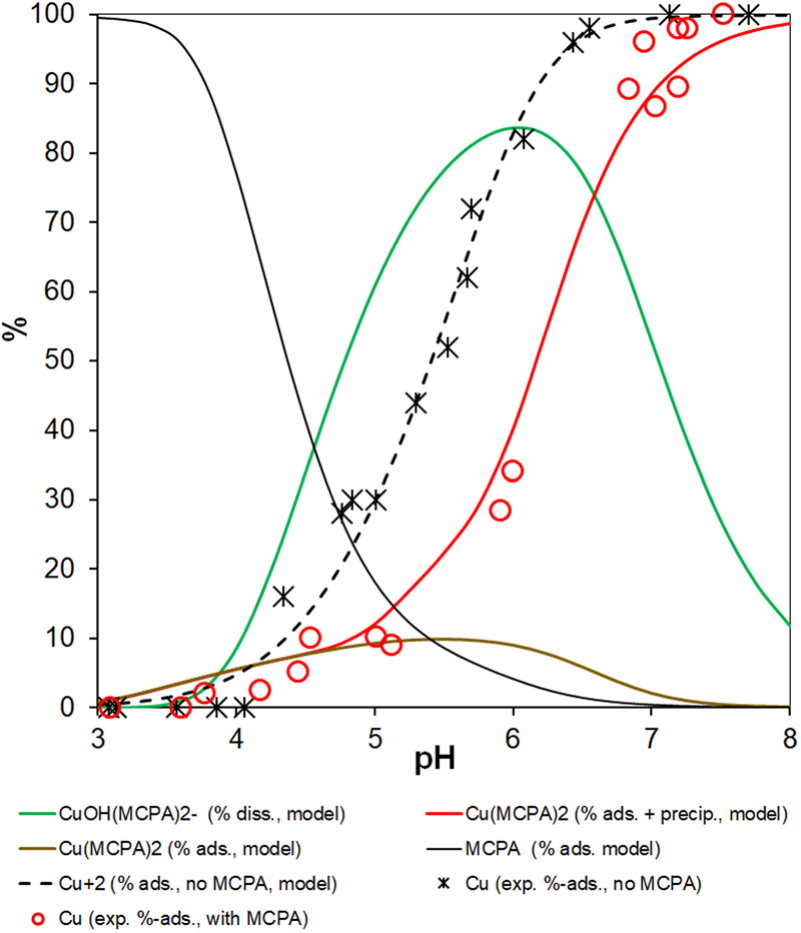
MCPA and Cu speciation in Cu–MCPA–goethite systems (5 g L^−1^ goethite, 0.5 mmol L^−1^ Cu and/or 1.0 mmol L^−1^ MCPA). The solid and dotted lines are the model curves. The asterisks are experimental data for the binary Cu–goethite system without MCPA, the red open dots are experimental data for the ternary system Cu–MCPA–goethite. The red curve is for polymerization and shows the percentage of the Cu(MCPA)_2_ complex that precipitated to give almost no dissolved Cu at pH > 7 in the batch equilibrium experiments.

The experimental details and data interpretation using an advanced adsorption model on basis of the charge distribution multi-site surface complexation (CD-MUSIC) approach are reported in the Supporting Information. Adsorption model curves were calculated using the VMINTEQ (version 3.1)^21^ speciation code with the thermodynamic data compiled in the Supplementary Information Tables S1 and S2. The results are shown in Fig. 1, where the black asterisks and dotted line represent first the experimental and modeled data for Cu adsorption by goethite (α-FeOOH) without MCPA. The percentage of Cu adsorbed increased from 10% to 90% over a narrow pH range. Under the experimental conditions used in the PET imaging experiments with artificial soil columns (5 g L^−1^ goethite, 0.5 mmol L^−1^ Cu), the adsorption edge was at pH_50_ = 5.4. On the other hand, pure MCPA is effectively adsorbed by goethite only at pH < 4 (black curve in Fig. 1). A steep increase in the amount of free anionic MCPA in solution above pH 4 enables to complex the Cu^2+^ (to >80% at pH 5.4, Fig. 1). The presence of a residual non-covered carboxyl group in a phenoxyalkanoic acid molecule allows the herbicide to form complexes with divalent and trivalent metal ions (e.g., Cu, Zn, Mn, and Al ions)^22^. Thermodynamic speciation calculations showed that the dominant dissolved Cu species above pH 4 is the monovalent deprotonated anion complex Cu(OH)(OOCR)_2_^−^, in presence even of equimolar fulvic and humic acids (Supplementary Information Table S1 and Fig. S1). The data for the ternary Cu-MCPA-goethite system (red open dots in Fig. 1) indicate that at pH 5.4, much less Cu is adsorbed than in the binary Cu-goethite system. The dissolved Cu-MCPA complex formed at pH > 4 outcompetes the surface complexation of the Cu^2+^ cation by goethite. The significant decrease in Cu adsorption at acidic soil pH values (pH 4–6) caused a shift in the Cu adsorption edge by one unit to pH_50_ = 6.4 (Fig. 1). While for glyphosate this complexation effect is counterbalanced by strong ternary inner-sphere surface complex formation^23^, this is obviously not the case for MCPA. No ternary Cu–MCPA inner-sphere surface complex formation has been found by EXAFS analysis (data not shown), likely because of no free functional groups left over as in case of glyphosate^24^. In presence of MCPA, only weak outer-sphere ternary surface complex formation occurred of the anionic Cu-MCPA moeity, contributing about 10% of the adsorbed Cu at pH 5.4 (brown curve in Fig. 1). This agrees with the hypothesis^17^ of a “water-bridging” (i.e., outer-sphere surface complexation) mechanism of MCPA adsorption to soil minerals mediated by Fe cations.

Finally, at pH > 6, both the dissolved MCPA and Cu concentrations decreased again in a stoichiometric molar 1:2 ratio. This decrease was found both with and without goethite, indicating that the loss was probably caused by the precipitation of solid Cu(MCPA)_2_^25^ rather than by Cu adsorption or tenorite (Cu(OH)_2_) precipitation. Despite the relatively high total concentration of 0.5 mmol L^−1^, the Cu is undersaturated with respect to tenorite $({{\rm{SI}}}_{{\rm{Cu}}{({\rm{OH}})}_{2}}\, < \,-\,2)$ within this pH range due to the strong MCPA complexation. Nonetheless, further hydrolysis at pH > 6 causes the complex to polymerize and form a light blue precipitate. Polymerization is accounted for in our speciation model by the formation of the oxo-bridged binuclear complex Cu_2_O_2_(OOCR)_4_^4−^, in agreement with recent results^26^ of X-ray structure analysis of the analogous compound [Cu_2_(H_2_tea)_2_(2,4,5-trichlorophenoxyacetate)_2_]·H_2_O. We hypothesize that the dimer thus formed may be the initial product of hydrolytic polymerization that is typical of many metal cations in circumneutral and alkaline aqueous solutions. Protonation equilibrium causes the strong pH-dependence of dimer formation, and the stability constant for the dimer matches quite well the pH-dependent solubility of Cu in presence of MCPA. For the red model curve in Fig. 1, we merged both this polymerization effect with the percentage adsorbed Cu(MCPA)_2_ at lower pH values to match best the experimental points over the pH range 3–8 studied. Since the Cu mobility in presence of goethite was found to be most enhanced by MCPA in between pH 5–6, it is within this pH window where the PET experiments with the artificial soil columns were deemed to become most interesting and successful.

### Molecular 4D PET Imaging

For the PET imaging experiments, artificial soil columns (inner diameter 40 mm and a height of 100 mm) were packed with a mixture of quartz sand (68 wt.-%) and silt (26 wt.-%), both coated with goethite (1 wt.-%), and clay particles (5 wt.-%). Preliminary batch equilibrium experiments using ^64^Cu-radiolabeled Cu(MCPA)_2_ solution with the column filling material were performed to reproduce the batch equilibrium adsorption experiment results (Supplementary Information Fig. S3). Four PET experiments were performed using two test column replicates. With the first column two experiments were performed consecutively using the reasonably non-reactive tracer solution of [^18^F]KF, another two with the second column using the reactive tracer solution of [^64^Cu]Cu(MCPA)_2_. The test column experimental conditions are listed in Table 1. Hydrodynamic properties for the two experiments with ^64^Cu calculated under the assumption of homogeneous column matrix properties and plug-flow like fluid dynamics are compiled in Table 2. The relative higher contribution of advection over diffusion, that interact to generate hydrodynamic dispersion, are characterized by a Peclet number *Pe* = 200 (Table 2).

**Table 1:** Summary of the four PET experiments with two different test columns.

Column #	1		2	
Experiment #	1	2	3	4
Purpose	Saturation with water	Non-reactive transport	Initial reactive transport	Equilibrated reactive transport
Tracer	[^18^F]KF	[^18^F]KF	[^64^Cu](Cu-MCPA_2_)	[^64^Cu](Cu-MCPA_2_)
Activity (MBq)	137	149	243	106
Pore fluid and inflow pH value	10mM KF, 1 mM NaNO_3_, pH 6.8	10 mM KF, 1 mM NaNO_3_, pH = 6.8	10 mM KF, 1 mM NaNO_3_, pH = 5.2	10 mM KF, 1 mM NaNO_3_, pH = 5.2
Carrier solution	10 mM KF, 1 mM NaNO_3_, pH = 6.8	10 mM KF, 1 mM NaNO_3_, pH = 6.8	0.8 mM Cu-MCPA_2_, 10 mM KF, 1 mM NaNO_3_, pH = 4.9	0.8 mM Cu-MCPA_2_, 10 mM KF, 1 mM NaNO_3_, pH = 4.9
Number of frames	37	38	67	65
Frame length (min)	12–40	12–40	12–120	12–120
Period (h)	16	21	215	191
Injected volume (mL)	91	123	680	735

**Table 2:** Exemplary geometrical and hydrodynamic parameter for the artificial soil column as used in the PET experiments #3 and #4 with the reactive [^64^Cu](Cu-MCPA_2_) tracer (suffix *e* = experimental data, *c* = calculated).

Parameter	Sign	Value column #3	Value column #4	Unit
Inner diameter^e^	*d*	40.0 ± 0.05	40.0 ± 0.05	mm
Colum length^e^	*l*	100.0 ± 0.05	100.0 ± 0.05	mm
Cross-sectional area^e^	*A*	12.57 ± 0.45	13.2 ± 0.45	cm^2^
Height of filling^e^	*h*	94.5 ± 0.05	90.1 ± 0.05	mm
Volume of filling^e^	*V*_c_	119 ± 6.0	114 ± 5.7	cm^3^
Dry mass of filling^e^	*m*_d_	228.3 ± 0.1	206.8 ± 0.1	g
Wet mass of filling^e^	*m*_w_	262.4 ± 0.1	239.4 ± 0.1	g
Total pore volume^e^	*V*_p_	34.1 ± 6.0	33.6 ± 5.7	mL
Bulk density of filling^e^	*ρ*_*B*_	2.66	2.66	g cm^−3^
Porosity^e^	*θ*	0.29 ± 0.05	0.30 ± 0.05	—
Pressure gradient^e^	Δ*p*	0.20 ± 0.01	0.20 ± 0.01	bar
Flow rate^e^	*Q*	100 ± 1	100 ± 1	μL min
Permeability^c^	*k*_f_	7.1·10^−8^ ± 0.7·10^−8^	5.9·10^−8^ ± 0.6·10^−8^	m s^−1^
Darcy permeability^c^	*K*	7.3·10^−3^ ± 0.7·10^−3^	6.1·10^−3^ ± 0.6·10^−3^	Darcy
Water flow velocity^c^	*v*_w_	4.5·10^−6^ ± 9·10^−7^	4.6·10^−6^ ± 9·10^−7^	m s^−1^
Interstitial velocity^c^	ν	—	1.6·10^−4 ± ^3·10^−6^	m s^−1^
Axial dispersivity^c^	*α*_L_	—	8.7·10^−4^ ± 9·10^−6^	m
Mean tracer velocity^c^	*v*_*t*_	2.7·10^−7^ ± 1·10^−9^	2.4·10^−7^ ± 1·10^−9^	m s^−1^
Peclet number^c,^*	*Pe*	—	200	—
Retardation factor^c,^**	*R*	17.3 ± 3.2	19.1 ± 3.6	—

*The Peclet number *Pe* was derived from the axial dispersivity of *α*_L_ = 8.7·10^−4^ m, the interstitial velocity of *v* = 1.6·10^−4^ m s^−1^, and a molecular diffusion coefficient for MCPA of *D*_m_ = 6.8·10^−10^ m^2^ s^−1^.

**The retardation factor was calculated as ratio between mean water and mean tracer velocities.

**Figure 2:**
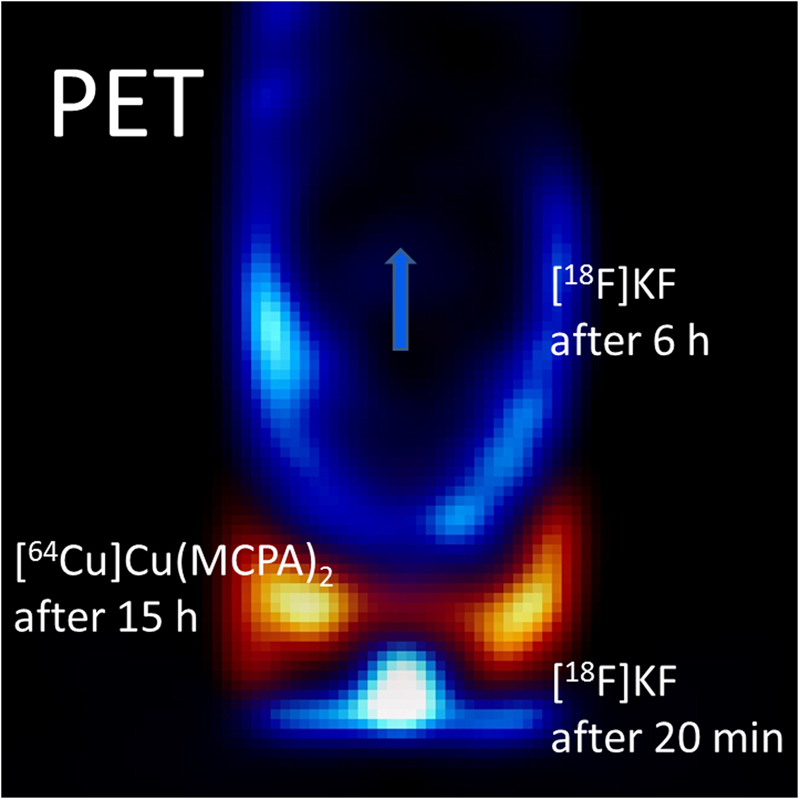
The snapshot shows an overlay of tracer propagation of both the conservative [^18^F]KF tracer experiment #2 (blue colors) and the reactive [^64^Cu]Cu(MCPA)_2_ tracer experiment #3 (orange colors) at different times. Comparison of the two frames of experiment #2 (20 min and 6 h after injection of the tracer pulse) with one frame of experiment #3 (15 h after injection of the tracer pulse) shows substantial retardation of the reactive tracer [^64^Cu]Cu(MCPA)_2_. Frame height is 85 mm, frame width is 40 mm, voxel size is 1.15 mm. A smoothing of the image data was applied to increase the signal-to-noise ratio, but causes reduction of the spatial resolution to 2–3 mm.

**Figure 3:**
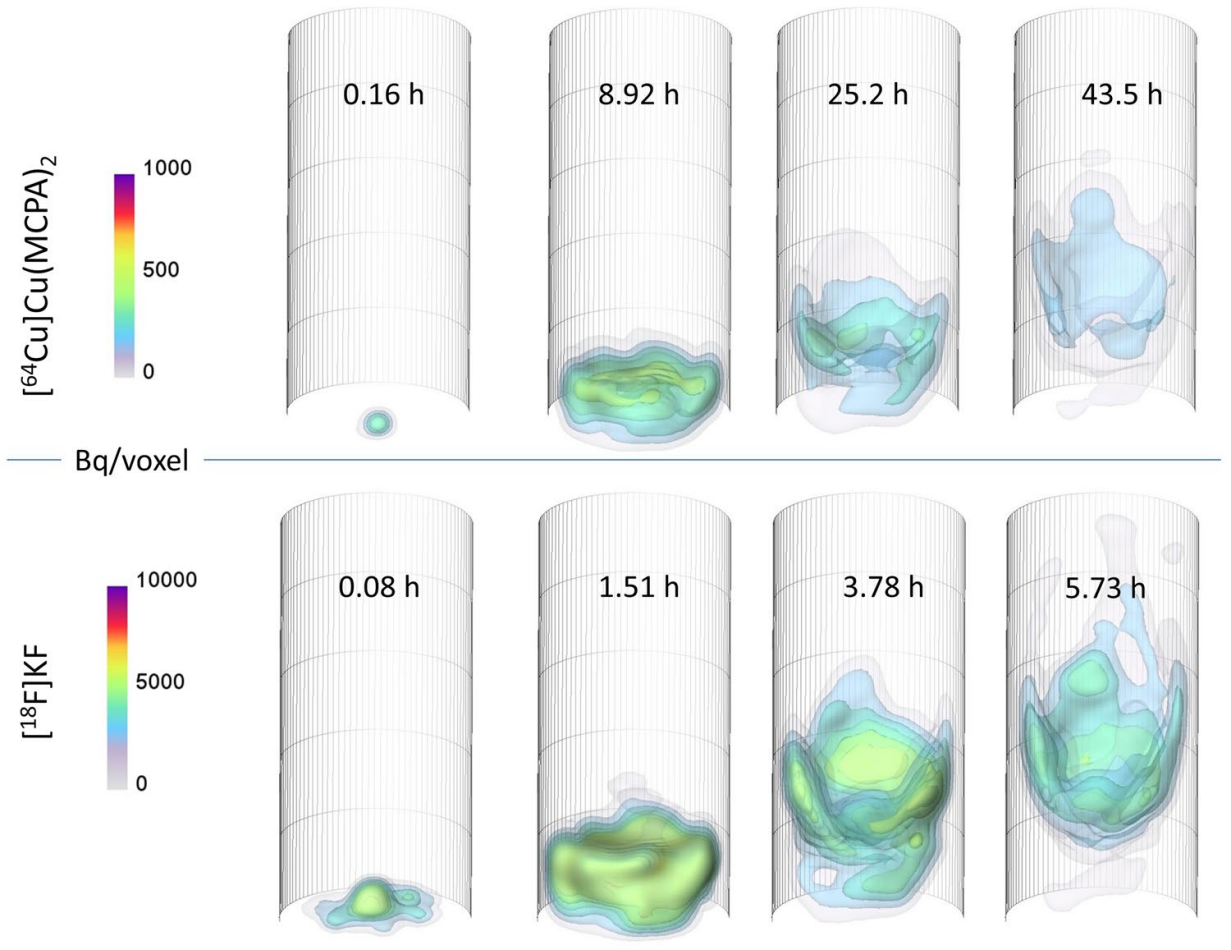
Time-resolved positron emission tomography (4D PET) images acquired during the propagation of a tracer pulse showing non-uniform flow phenomena in an artificial soil column. The tracer distributions are shown as overlaid isosurfaces. The top four images show retarded transport of ^64^Cu radiolabeled Cu-MCPA complex, and the lower images show the transport of ^18^F-labeled KF as a reasonably conservative reference solute. The selected images represent comparable stages of propagation but were taken at different times. Full movies of all four test column experiments are downloadable as mpeg-4 video file from Supplementary Information.

A snapshot shows for different times an overlay of tracer propagation of both the non-reactive [^18^F]KF tracer experiment #2 (blue colors in Fig. 2) and the reactive [^64^Cu]Cu(MCPA)_2_ tracer experiment #3 (orange colors in Fig. 2). Comparison of the two frames of experiment #2 (20 min and 6 h after injection of the tracer pulse) with one frame of experiment #3 (15 h after injection of the tracer pulse) suggests substantial retardation of the reactive tracer. Only about 66 h into experiment #3 the ^64^Cu tracer started to breakthrough. In the replicate experiment #4, first tracer breakthrough was found already at 22 h. The normalized (i.e., decay-corrected) activity continued to increase until it reached a maximum at 98 h for experiment #3 (77 h for experiment #4). A retardation factor of 17.3 ± 3.2 was estimated from the difference in ^18^F and ^64^Cu tracer velocities for experiment #3. For the replicate PET experiment #4 the retardation factor was slightly higher at 19.1 ± 3.6. These differences were likely due to some instability of the pH value within both test columns (inflow solution pH 5.2, outflow solution pH 5.9). This suggests that the overall reproducibility might have been somewhat limited by the development of a pH gradient within the test columns, in particular as the amount of Cu adsorbed increased slightly from 10% to 30% in the pH range 5–6 (Fig. 1). Preloading with the Cu-MCPA complex for the replicate experiment #4 might have somewhat accelerated the initial breakthrough along the preferential pathways, which might explain the earlier break through albeit at similar overall retardation behavior. The migration of the radiolabeled molecules through one of the soil columns is shown as 3D screenshots at four time points in Fig. 3. Time-lapse movies of the reactive transport of ^64^Cu-labeled Cu-MCPA complex versus the non-reactive transport of ^18^F-labelled KF solute, each produced from 37–67 PET frames from all four cores studied, are available in the Supplementary Information mpeg-4 video file.

**Figure 4:**
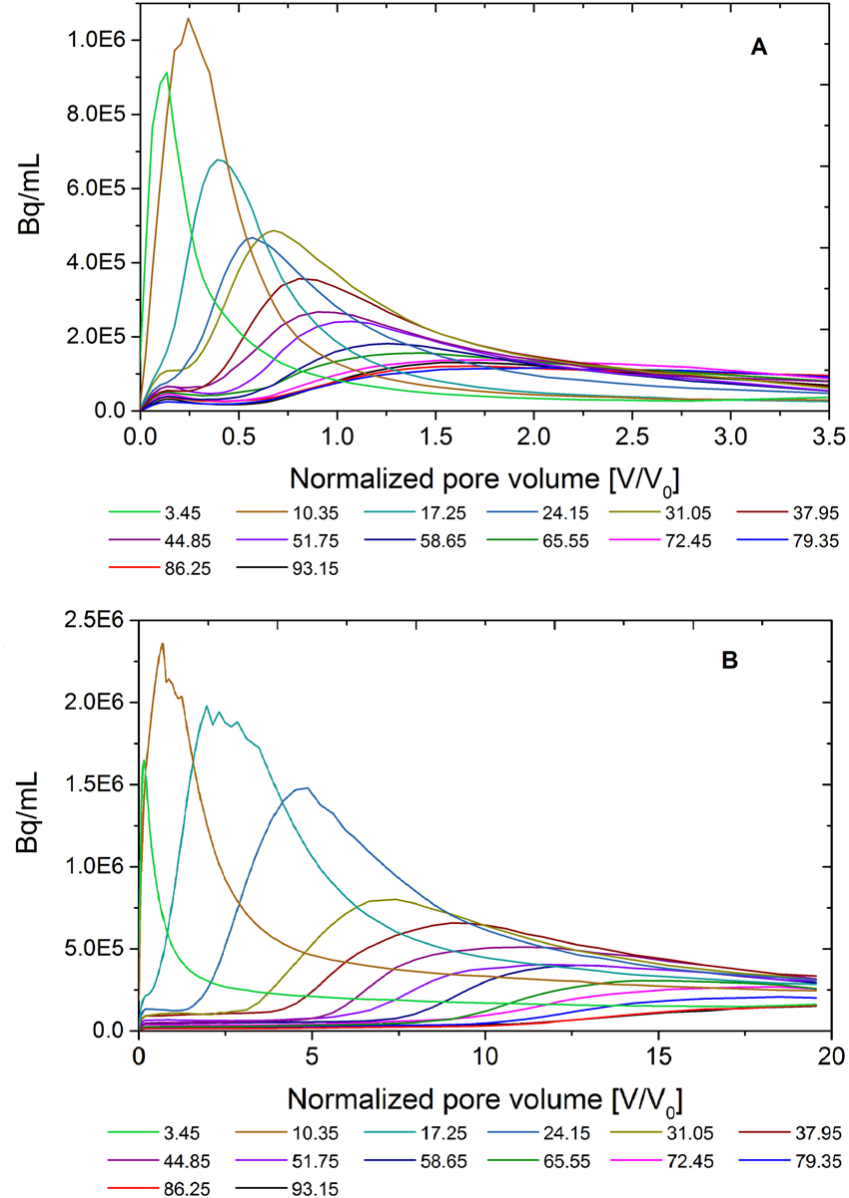
Range of virtual breakthrough curves calculated as average over inner cross-sectional slices with distances of 6.9 mm each along the column, for (**A**) the conservative tracer [^18^F]KF in column of experiment #2, and (**B**) the reactive tracer [^64^Cu]Cu(MCPA)_2_ in experiment #3.

All possible efforts were made to ensure that the column was packed homogeneously, but no plug-flow of both non-reactive and reactive tracers occurred in the repacked soil column. Rather than a piston-like displacement of the background fluid and homogeneous upward advection through the column, the tracer pulse dispersed longitudinally to form a notable asymmetry to the transport plume. Preferential flow paths developed in the form of pronounced fingering at the column edges and lower permeability zone with a transport lag in the column center. The pattern of this fingering is not reproducible and changes from one column to the other (cf. the video with both test column PET imaging results in the Supporting Information). Despite the fact that no heterogeneities were intentionally included into the column, both the conservative ^18^F and reactive ^64^Cu plume evolutions suggested a macroscopic non-uniform flow behavior. This considerably decreased the effective flow process volume and increased the effective transport velocity relative to homogeneous flow conditions. When the tracer front reached about half of the column height, the activity apparently wears off or seems to disappear into the bulk packing. However, this was caused by known decay, dilution and dispersion behavior into the larger volume, and all these effects ultimately decreased the activity concentration to below the noise level and detection threshold. Our hypothesis is that heterogeneous displacement of the clay particle content may have played a role in development of a fluid flow rather than reaction heterogeneity. Clay particle displacement has already been evidenced by X-ray tomography data^27^ for another such case earlier, but a discussion of this is beyond the scope of this paper. The consequence of the asymmetry in flow is shown with virtual breakthrough curves calculated over equidistant cross sections along the column (Fig. 4). The curve tailing increased significantly along the length of the column as typical for non-uniform pulse flow rather than for flow within a sediment sample expected to be homogeneous.

## Discussion

Applying MCPA to acidic soils that have previously been amended with Cu-containing fungicides (e.g., the Bordeaux solution) may cause remobilization of at least a portion of the Cu in the soil. This hypothesis was supported by surface complexation modeling and, first for a reactive tracer, by molecular PET imaging using a repacked test column. PET imaging enabled time-lapse movies of the ^64^Cu labelled Cu-MCPA complex migration at relatively high spatial and temporal resolution to be recorded. This effort was supported by specially adapted data correction methods that have been under development for a decade. They now allow quantitative tomograms with an unprecedented image quality sustained at a level allowing the capture of the spatiotemporal evolution of 3D reactive tracer migration in an opaque soil column. The molecular dynamic PET imaging experiments indicate that it takes just a few days for Cu-loaded herbicide to break through 10 cm of an acidic (pH 5–6) soil. The flow plumes of the reactive tracer were quite heterogeneous and not reproducible by replicate test columns. Many mechanisms can cause local transport heterogeneity, including (i) inhomogeneous solid phase distribution or re-distribution during flow, or (ii) inhomogeneous reaction pathways. Our PET results with the non-reacting ^18^F radioisotope showing nearly the same non-uniform flow patterns evidenced that inhomogeneous fluid flow rather than inhomogeneous reaction pathways was the main source of heterogeneity.

It should be noted that, in contrast to the conditions of this laboratory study, retardation and degradation processes occur simultaneously under field conditions. Clearly, the process of determining Cu-MCPA risk beyond the lab scale should be an analysis of true risk documented in field. This lab-based study provides an alarming hypothesis at best, which warrants tests to be performed using different natural soils and inherently different microbial populations using lysimeters under natural field conditions. Such tests will allow estimation of reliable microbial degradation rates and, hence, the GUS index parameters. While MCPA has a relatively short half-life of a few months because it is quickly mineralized by microbes in soil^28,29^, no such data are yet available for the Cu-MCPA complex. It is known that such complexation not only impacts the mobility of trace metals but also the degradation (both abiotic and biotic) of glyphosate, and MCPA might become less degradable as well. The tests should also involve evaluating the promising new ionic liquid forms of MCPA. In a recent field study it was shown that using an alternative ionic liquid formulation of [Etq O-12]-MCPA or DDA-MCPA did not affect metal uptake by spring barley^30^. This is because the structure of the novel ionic liquid herbicide made it practically impossible for the herbicide molecules to form complexes with metal cations.

## Methods

### Repacked Artificial Soil Column Experiments

Batch equilibrium adsorption experiments were performed as further detailed in the Supporting Information and saturated laboratory-scale column experiments, both with same artificial soil mineral material. The porous matrix of the column filling was a mixture of 68 wt.-% laboratory-grade quartz sand (Haltern H33, mean grain size 0.26 mm; Quarzwerke, Frechen, Germany) and 26 wt.-% quartz silt (Millisil Haltern W11, mean grain size 40 μm, with a lower cutoff of 36 μm achieved by dry sieving; Quarzwerke). The sand/silt mixture was pre-coated with 1.0 wt.-% of goethite nanoparticles (Bayferrox 910 Z, LANXESS, Köln, Germany), which provides for the only reactive material in both the batch equilibrium adsorption and the soil column experiments. Soil organic matter was not added to avoid triggering microbial degradation, and because MCPA is not bound significantly by SOM in presence of goethite^19^. Illite clay mineral (5.0 wt.-%) was admixed merely to mimic the water retention capabilities typical of natural soils. The sediment constituent mixture was slurry-packed into polymethyl methacrylate columns (inner diameter of 41 mm and a height of 100 mm) within a glove box under a CO_2_ atmosphere, to avoid trapping residual N_2_ microbubbles in the pore microstructure. The entrance cap was covered with 105 μm tetrafluoroethylene gauze to minimize the dead space and to allow an optimum injected fluid distribution over the entire cross-sectional area of the column. Two columns were packed, one for a replicate ^18^F experiments #1 and #2, another one for a replicate ^64^Cu experiments #3 and #4 (Table 1).

The columns were preconditioned with 1 mmol L^−1^ NaNO_3_ background electrolyte solution of pH 5.2 for one week. The solution was supplied to the column from bottom up via polytetrafluoroethylene capillaries by an HPLC pump at a constant fully saturated volumetric flow rate of 100 μL min^−1^. The flow rate was relatively low (i) to allow for equilibration between the reactive tracer and substrate particles in the artificial soil column, and (ii) to maintain a residence time of a few hours to allow breakthrough of the tracer to be detected within few times of its half-life (^64^Cu T_1/2_ = 12.7 h and ^18^F T_1/2_ = 1.83 h). The two applied positron emitting radionuclides ^18^F and ^64^Cu were produced on-site using a cyclotron (Cyclone 18/9, IBA Radiopharma Solutions, Louvain-la-Neuve, Belgium) as described in Supplementary Information Fig. S3. The workflow scheme of the PET experiment is shown in Supplementary Information Fig. S4. A small volume (5 mL) of tracer solution at pH 4.9 containing 10 mmol L^−1^ KF and 1 mmol L^−1^ NaNO_3_ was prepared for use as the carrier and background solution. In the first experiment column series, the KF carrier was labeled with about 137 or 149 MBq of ^18^F tracer. In the second experiment series  using another column, the carrier solution contained 0.8 mmol L^−1^ Cu(MCPA)_2_ labeled with about 243 or  106 MBq ^64^Cu tracer (Table 1). In each experiment, 4 mL was applied to the column and 1 mL was retained as a back-up to correct for decay. The tracer solution was injected using a syringe pump at continuous flow rate in between pure background electrolyte injection as a rectangular Dirac pulse of tracer solution for 40 min. The outlet solution passed a continuous flow-through scintillation gamma counter (GABI Star (Elysia-Raytest). The in-line measured activity was lastly confirmed on discontinuous samples with a gamma counter (Wizard 3; Perkin-Elmer, Waltham, MA, USA). Both counters were equipped with NaI(Tl) detectors. They allowed to perform material balances (Bq in vs. Bq out) in particular to support the use of the conservative tracer ^18^F. Tracer breakthrough curves could therefore be determined by monitoring the effluent activity without interrupting the flow for up to about eight half-lives of the tracer. Both experiments were repeated with same columns (^18^F experiments #1 and #2 with the first column, ^64^Cu experiments #3 and #4 with the second column, Table 1).

### Positron Emission Tomography (PET)

The principles of PET applications to geoscientific process studies have previously been described in detail^31^. Our PET scanner (ClearPET; Elysia-Raytest, Straubenhardt, Germany) exploits the physical resolution limit with voxel sizes of 1.15 mm^3^ at a maximum field-of-view of 136 mm (145 × 145 × 95 voxels), fully suitable for core-scale process tomography^32^. The repacked soil columns were rigidly mounted vertically on a slide in the center of the PET gantry (Supplementary Information Fig. S5). The data correction and image reconstruction algorithms used were based on a workflow^33^ with the Open Source STIR library. Unlike with SPECT, it is not necessary to stop flow intermittently during the experiment to facilitate the collection of the imaging data at high resolution without blurring effects. However, unlike in biomedical applications, particular attention had to be paid to attenuation and scattering of the annihilation photon radiation because a substantial proportion is lost as the radiation passes through a dense geomaterial. Attenuation correction was made easier by the regular cylindrical core geometry and the mostly homogeneous and well-defined density with low variation in mass attenuation coefficients. It was more difficult to correct scatter for the effective Compton absorption process, which is dominant at the relevant energy range in dense geomaterials. The default ClearPET scatter correction algorithm was inappropriate, but the single-scatter simulation algorithm in the STIR 3.0 library was found appropriate^34^ when supported by Monte Carlo simulations as a forward-modeling option for tomographic inversion. A thorough bin-wise normalization and absorption correction procedure and filtering during the iterative reconstruction improved the image quality. The data were subdivided into 30–60 frames, the frame length increasing from 12 to 120 min through the experiment to account for tracer decay. The images were decay-corrected, and total activity was calibrated with respect to the known administered activity, allowing the tracer activity concentration per voxel and selected time step to be quantified. The AVIZO FIRE software package was ultimately used to produce time-lapse presentations of the activity distribution and semi-transparent iso-surfaces in 3D.

## Electronic supplementary material

Supplementary Information

Video of PET movies showing time-lapse Cu-MCPA migration in 3D within the test columns
