# Supplementary material for: Time-lapse 3D imaging by positron emission tomography of Cu mobilized in a soil column by the herbicide MCPA
Source: Sci Rep. 2018 May 4;8:7091. doi: 10.1038/s41598-018-25413-9 (PMC5935730; doi:10.1038/s41598-018-25413-9)
Supplement: Supplementary file 1 — Supplementary Information [file 41598_2018_25413_MOESM1_ESM.pdf]

Time-lapse 3D imaging by positron emission tomography of Cu mobilized in a soil column by the herbicide MCPA

Johannes Kulenkampff[1,*], Madeleine Stoll[1,2,3], Marion Gründig[1], Alexander Mansel[1,4], Johanna Lippmann-Pipke[1,5], and Michael Kersten[2]

[1]Helmholtz Zentrum Dresden-Rossendorf (HZDR), Institute of Resource Ecology, Research Site Leipzig,, Permoserstr. 15, 04318 Leipzig, Germany

[2]Geosciences Institute, Johannes Gutenberg University, J.-J. Becherweg 21, 55099 Mainz, Germany

[3]Present address: Institute of Nuclear Waste Disposal, Karlsruhe Institute of Technology, Hermann-von-Helmholtz-Platz 1, 76344 Eggenstein-Leopoldshafen, Germany

[4]Helmholtz-Institute Freiberg for Resource Technology, Metallurgy and Recycling Division, Chemnitzer Str. 40, 09599 Freiberg, Germany

[5]Present address: Federal Institute for Geosciences and Natural Resources (BGR), Rock Characterization for Storage and Final Disposal, Stilleweg 2, 30655 Hannover, Germany

*Corresponding Author, Email: j.kulenkampff@hzdr.de

Supporting Information

1. MCPA form dissolved Cu complexes p. S2
2. Batch Equilibrium Adsorption Experiments p. S3
3. Setup of the CD-MUSIC surface complexation model p. S4
4. Cu-64 radiotracer production using a cyclotron p. S6
5. The PET experiment p. S7
6. References p. S9

10 Pages, 6 Figures, and 2 Tables

MCPA form dissolved Cu complexes. At ambient pH values, phenoxyalkanoic acid herbicides are relatively weakly retarded in soil. The extent of sorption is negatively correlated with soil pH and positively correlated with organic matter content (Paszko et al., 2016). The acidity constants pK_a decrease in the order 2,4-DB > dichlorprop-*p* > 2,4-D and MCPB > mecoprop-P > MCPA. Consequently, the fraction of the anionic forms of these herbicides and hence hydrophilicity at ambient soil pH values increases in the same order. In MCPA (pK_a = 3.09 ± 0.02), one methyl group of the phenoxy moeity is replaced with a chlorine atom. The polarity and aqueous solubility of its sodium–potassium salt is thereby increased (Haberhauer et al., 2000, 2001). Due to its low pK_a value and hydrophilicity, it has a higher affinity to more polar dissolved fulvic acids and a lower affinity to particulate humic acids (Iglesias et al., 2009; Paszko et al., 2016). Similarly as with the latter acids, trace metals can be complexed by the carboxyl group of MCPA. X-ray absorption spectroscopy showed that the Cu(II) cation in the $Cu(MCPA)_2$ complex is six-coordinated (CuO_6) and formed a Jahn–Teller distorted octahedron (Klepka et al., 2012). The inner coordination sphere of the square planar portion around the Cu(II) cation contained two oxygen atoms from water molecules and two oxygen atoms from the carboxylate groups of the two monodentate-bound ligands, and the axial positions were occupied by ether oxygen atoms of two centrosymmetric *trans*-related *bis*-chelate ligands. From the results of potentiometric titration experiments, Masbouh et al. (2006) suggested that at least one of the two water molecules is hydrolysed, giving the ternary anionic complex $Cu(OH)(OOCR)_2^-$ with a negative charge. The Cu complexation by MCPA chelate is quite strong in comparison to other organic acids to be found in soil solution (Table S1). For example, a simulation with 10 mmol/L Cu in presence of each 2 mmol/L of MCPA and 1 mmol/L each of fulvic (dissolved) and humic (solid) acid using the "Stockholm Humic Matter (SHM)" model as default in Visual MINTEQ 3.1 code (Gustafsson, 2016) demonstrates importance of MCPA complexation in the pH range 4 - 8 (Figure S1).

Table S1. Complexation constants of Cu-MCPA in comparison with other dissolved organic Cu complexes like with fulvic acid (HFA). Data for Cu-MCPA from Masbouh et al. (2006), all other data from Visual MINTEQ 3.1 database (Gustafsson, 2016).

Species	Cu^{+2}	H^+	$MCPA^{-1}$	HFA	$Glycine^{-1}$	$Citrate^{-3}$	$GlyPhos^{-2}$	log*K*
H-MCPA	0	1	1	0	0	0	0	3.1
$CuOH(MCPA)_2^{-1}$	1	-1	1	0	0	0	0	4.8
$Cu_2O_2(MCPA)_4^{-4}$	2	-4	1	0	0	0	0	-0.3
$(FA)_2Cu(aq)$	1	-2	0	2	0	0	0	-5.8
$(HA)_2Cu(s)$	1	-3	0	2	0	0	0	-6.0
$Cu\text{-}(Glycine)_2$	1	0	0	0	2	0	0	15.7
$Cu\text{-}Citrate^{-1}$	1	0	0	0	0	1	0	7.6
$CuH\text{-}Citrate^0$	1	1	0	0	0	1	0	11.0
$CuGlyPhos^0$	1	0	0	0	0	0	1	3.7

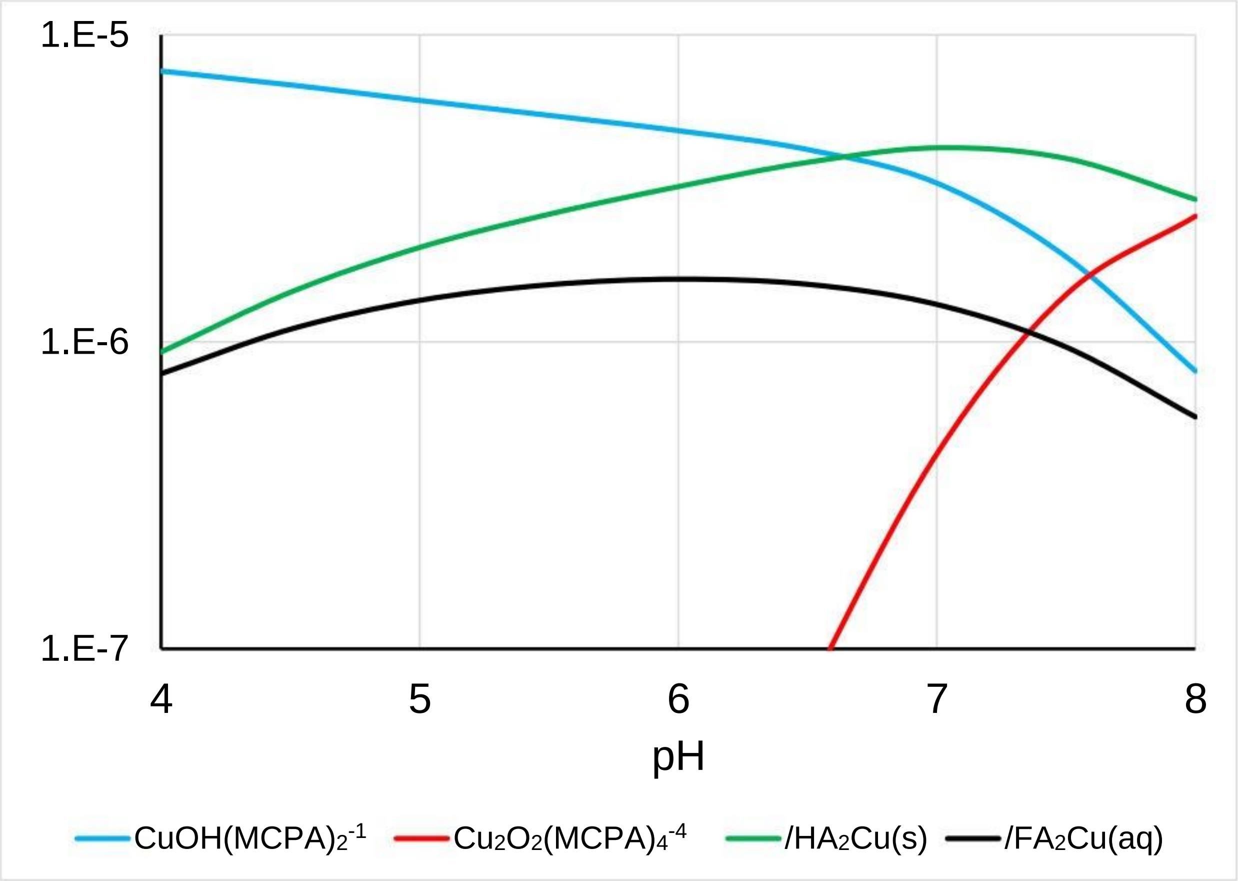

Figure S1. Simulation with 10 µmol L^{-1} Cu in presence of 2 mmol L^{-1} of MCPA and 1 mmol L^{-1} (site density) each of fulvic (/FA) and humic (/HA) acid using the Visual MINTEQ 3.1 code with complexation constants compiled in Table S1.

Batch Equilibrium Adsorption Experiments. The synthetic goethite used was Bayferrox 910 Z (LANXESS, Köln, Germany), which is a base material used in fixed-bed adsorption columns in waterworks worldwide (e.g., the Sorb 33™ system used by Severn Trent in the USA). MCPA (99.0% pure; Sigma-Aldrich, Gillingham, UK) was purified by recrystallizing it from a 1:1 mixture of water and ethanol. In the first suspension batch series, 30 mL of the $Cu(MCPA)_2$ stock solution was poured into each of a series of 50 mL polypropylene centrifuge tubes. The mixtures in 10 tubes were adjusted to between pH 3 and 8 by adding 0.1 mol L^{-1} CO_2-free HNO_3 or NaOH dropwise. Other series of tubes were prepared each at 10 different pH values, but dry goethite was added to give a solid/solution ratio of 5 g L^{-1} when 30 mL of either the Cu or $Cu(MCPA)_2$ stock solutions were added. All the tubes were allowed to equilibrate with shaking for 48 h at 25 °C in a dark room to avoid photodegradation of MPCA. Preliminary kinetic studies showed that adsorption equilibrium was reached within 48 h (data not shown). Each tube was centrifuged at 3000 g for 20 min, and the supernatant was passed through a 0.2 µm Minisart hydrophilic membrane filter (Sartorius, Göttingen, Germany). The pH of each supernatant was measured using a pH meter equipped with a WTW Inolab Level 2P combination electrode (Xylem Analytics, Weilheim, Germany), which had been calibrated using CertiPur buffers at three different pH values (Merck). The total dissolved Cu concentration was measured using a Series 7700 inductively coupled plasma mass spectrometer (Agilent Technologies, Santa Clara, CA, USA).

Setup of the CD-MUSIC surface complexation model. Using a multisite surface complexation model requires information about the proton affinities of the various types of surface groups on all relevant crystal planes of the adsorbent. The needle-like goethite crystals have a rather high aspect ratio, so are dominated by the basal prism (110) surfaces. The site density for the singly coordinated –OH groups and triply coordinated μ_3-OH groups relevant to proton binding has been found to be (3.0 ± 0.3) nm^{-2} for such surfaces (Hiemstra and Van Riemsdijk, 2006). In the 1-pK approach used, both types of negatively charged surface hydroxyl groups ($FeOH^{-0.5}$ and $Fe_3O^{-0.5}$ in Table S2) become positively charged to +0.5 valence units (v.u.) by binding a proton. The proton affinity constants for both sites (pK_{H1} and pK_{H2}) were set to equal the goethite pH_{pzc}. An adsorbate, particularly an organic molecule, is large compared with a proton, so the charge was distributed over the electrical double layer to give a charge distribution multisite surface complexation (CD-MUSIC) model. In the extended Stern (three-plane) approach, the charges of the surface reactive functional groups of the adsorbate molecule are distributed over the surface plane (0-plane) and the first Stern plane (1-plane, Table S2). The precise location of the coordinating ligand charge at the solid–water interface is not explicitly known, and must be derived from structural information at the molecular level (EXAFS data). The adsorption model was parameterized using the speciation code VMINTEQ v3.1 coupled with the parameter fitting code PEST (Gustafsson, 2016). Activity corrections were made to the stability constants for the aqueous species using the Davies equation. The aqueous speciation database was extended using the dissolved MCPA-bearing species constants (Table S2). The background electrolyte ions can form ion pairs with both the singly and triply coordinated surface sites. The presence of the $NaNO_3$ background electrolyte was therefore considered using corresponding surface reactions with the intrinsic equilibrium reaction constants fixed at $\log K_{Na} = \log K_{NO_3} - \log K_{H1,2} = -0.6$ for symmetrical behavior (Lützenkirchen et al., 2008).

Table S2. Parameters used in the CD-MUSIC model of the binary Cu–goethite and MCPA–goethite systems and the ternary Cu–MCPA–goethite adsorption systems derived from fitting batch equilibrium experiment data

Species	$FeOH^{-0.5}$	$Fe_3O^{-0.5}$	H^{+1}	Na^{+1}	NO_3^{-1}	$MCPA^{-1}$	Cu^{+2}	Δz_0	Δz_1	Δz_2	log$K$
≡FeOH–H	1	0	1	0	0	0	0	1	0	0	6.8
≡Fe_3O–H	0	1	1	0	0	0	0	1	0	0	6.8
≡FeOH–HNO_3	1	0	1	0	1	0	0	1	-1	0	6.2
≡Fe_3O–HNO_3	0	1	1	0	1	0	0	1	-1	0	6.2
≡FeOH–Na	1	0	0	1	0	0	0	0	1	0	−0.6
≡Fe_3O–Na	0	1	0	1	0	0	0	0	1	0	−0.6
≡FeOH–Cu	1	0	0	0	0	0	1	0.84	1.16	0	8.7
≡FeOH–CuOH	1	0	-1	0	0	0	1	0.84	0.16	0	0.7
≡$FeOH_2$–$MCPA_{OS}$	1	0	1	0	0	1	0	1	-0.5	-0.5	9.2
$(≡FeOH)_2$–$MCPA_{IS}$	1	0	2	0	0	1	0	0.55	0.45	0	16.3
≡$FeOH_2$–$CuOHMCPA_{OS}$	1	0	0	0	0	1	1	1	-0.5	-0.5	15.0

Previously published models were used for both binary adsorption systems Cu–goethite and MCPA–goethite. The CD-MUSIC model was first used to calculate Cu adsorption considering bidentate inner-sphere Cu complexation with the singly coordinated surface sites of the goethite and allowing for hydrolysis according to reactions (3) and (4):

$$2{\equiv}FeOH^{-0.5} + Cu^{+2} \Leftrightarrow {\equiv}(FeOH)_2\text{-}Cu^{+1} \quad (3)$$

$$2{\equiv}FeOH^{-0.5} + Cu^{+2} + H_2O \Leftrightarrow {\equiv}(FeOH)_2\text{-}Cu(OH)^0 + H^+ \quad (4)$$

The corresponding charge distribution as suggested by Weng et al. (2008) is shown in Table S2, and the surface complexation constants were fitted to the experimental binary Cu–goethite system data from this study, also shown in Table S2, with quite good match (R = 0.99). The model results indicated that the hydrolyzed bidentate surface species dominated the adsorption edge and the non-hydrolyzed Cu surface species contributed only to tailing of the adsorption edge toward acidic pH values. The MCPA ligand can form both outer-sphere and inner-sphere monodentate complexes. In inner-sphere complexation, a carbonyl oxygen atom exchanges with a protonated surface hydroxyl group to release a water molecule according to reaction (5):

$$\equiv FeOH^{-0.5-} + H^+ + RCOOH \Leftrightarrow {\equiv}FeO\text{-}HOCR^{0.5+} + H_2O \quad (5)$$

The carboxylic group act as a monodentate ligand towards Fe(III), thereby leaving the non-coordinated carboxylic oxygen free to bridge through hydrogen bonding to either water molecules in the bulk solution, or to water molecules on the surface as can be seen in molecular dynamics movies (Kersten et al., 2014).

The adsorption reaction for the outer-sphere surface complex of the anionic MCPA molecule is shown in reaction (6):

$$\equiv FeOH^{-0.5} + RCOO^- + H^+ \Leftrightarrow {\equiv}FeOH_2^{0.5+}\,OOCR^- \quad (6)$$

The charge distribution for this monodentate inner-sphere complex of the neutral MCPA molecule was explained in detail previously (Kersten et al. 2014, Table S2). The monodentate inner-sphere surface complex of the protonated MCPA was dominant in the acidic pH range. Since MCPA adsorption was largely unaffected by $NaNO_3$ concentrations (10-100 mM), the contribution of this outer-sphere complex was insignificant and responsible only for some tailing of the adsorption edge toward the neutral pH range. Similarly, outer-sphere surface complex formation was assumed for the ternary complex, facilitated by the co-adsorption of one proton to render the surface charge positive, as shown in reaction (7):

$$\equiv FeOH^{-0.5} + Cu(OH)OOCR- + H^+ \Leftrightarrow {\equiv}FeOH_2^{0.5+}\,Cu(OH)OOCR^- \quad (7)$$

A good fit to the data was found if the charge on the ternary complex was not kept fully in one plane but was equally distributed over all three planes, leading to CD coefficients of $\Delta z_0 = 1$ (surface protonation), and $\Delta z_1 = \Delta z_2 = -0.5$ (Table S2). No inner-sphere surface complexation was assumed for this ternary complex because of steric hindrance. Any attempts to trace inner-sphere complexation using EXAFS failed (data not shown), and it was also not necessary to invoke such complex formation to improve the fit quality.

Cu-64 radiotracer production using a cyclotron. The radionuclide ^{64}Cu ($T_{1/2}$ = 12.7 h) was produced by means of the nuclear reaction $^{64}Ni(p,n)^{64}Cu$ (Szelecsényi et al., 1993). The target was prepared by electrodeposition of metallic [^{64}Ni]Ni (isotope enrichment for ^{64}Ni 99.09%, STB Isotope, Germany) from a 0.22 M $(NH_4)_2SO_4$ solution at pH 9, a voltage of 2.60 V and a current of 25 mA on a gold disc (Avila-Rodriguez et al., 1997). A target thickness of 38.8 mg/cm^2 was achieved. The Ni deposit was covered with a 100 µm thick Al foil to avoid Ni loss during the irradiation. Routine production runs of ^{64}Cu were performed at an initial energy of 12 MeV protons and at currents of ~20 µA lasting for 3-5 h at the Leipzig cyclotron Cyclone® 18/9 (IBA Molecular, Belgium) equipped with a COSTIS® solid target system (IBA Molecular, Belgium, see Fig. S5 below). The separation of ^{64}Cu from the target material ^{64}Ni was performed by anion exchange chromatography (Thieme et al., 2012). The irradiated Ni deposit was dissolved in boiling 12.5 M HCl, and the received solution was evaporated to dryness. The residue was re-dissolved in 6 M HCl (target solution). The anionic exchanger AG® 1-X8 resin (BIO-RAD, USA) was used to separate the target material ^{64}Ni from the radionuclide ^{64}Cu. The target solution was loaded on the resin column (5 cm in length, 0.5 cm in diameter) at a flux of 0.25 ml/min. The column was washed ten times with 6 M HCl (Ni fractions). These fractions were combined and the solution was evaporated to dryness. The residue was fumed two times with 18 M H_2SO_4, and the remaining [^{64}Ni]$NiSO_4$ was dissolved in 0.22 M $(NH_4)_2SO_4$ with a few drops of 13.5 M NH_4OH at pH 9 for the next electrodeposition. The ^{64}Cu was eluted from the column for twenty column volumes with 0.1 M HCl at a flux of 0.1 ml/min (Cu fractions). The combined Cu fractions were evaporated to dryness and the n.c.a. (no carrier added) ^{64}Cu was dissolved in the desired mineral acid (in this case, 0.1 M H_2SO_4). The ^{64}Cu activity per volume was measured with an automated γ-counter detector (1480 WIZARD 3”, PerkinElmer, USA), which was calibrated with a ^{152}Eu standard solution (PTB 443-88, PTB, Germany).

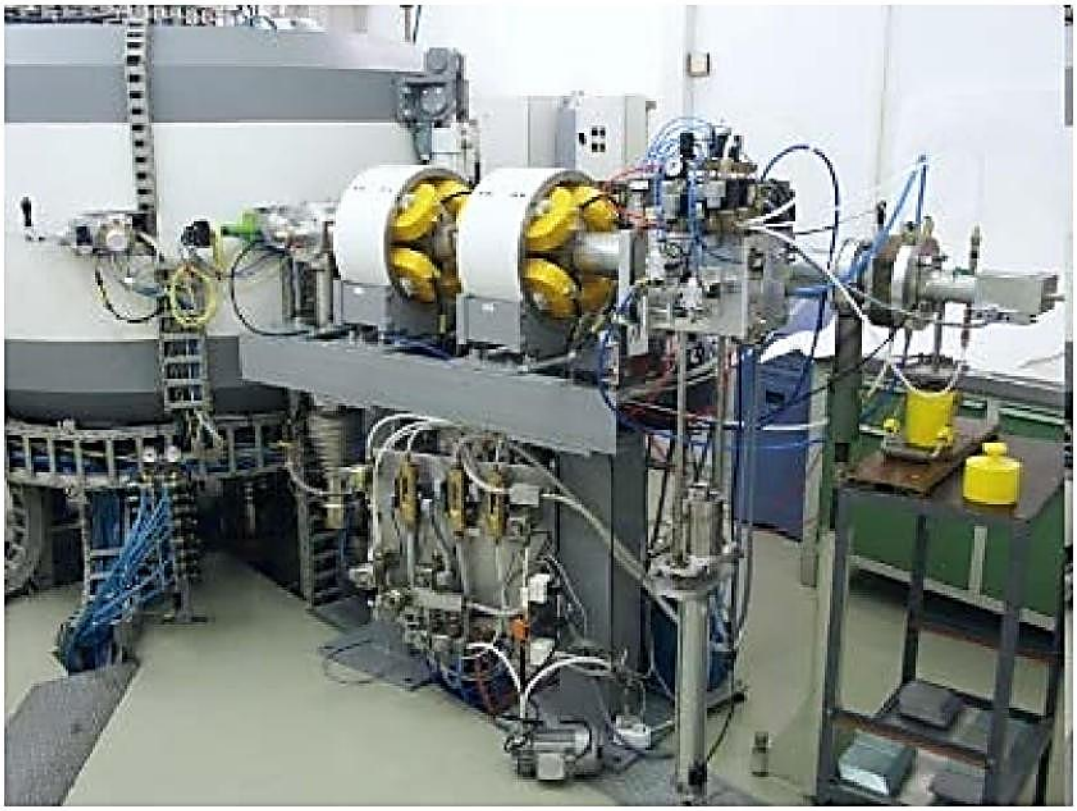

Figure S2. View inside the cyclotron of the HZDR Research Site Leipzig (Cyclone 18/9, IBA Radiopharma Solutions, Belgium). More information on the cyclotron can be found at: http://www.iba-radiopharmasolutions.com.

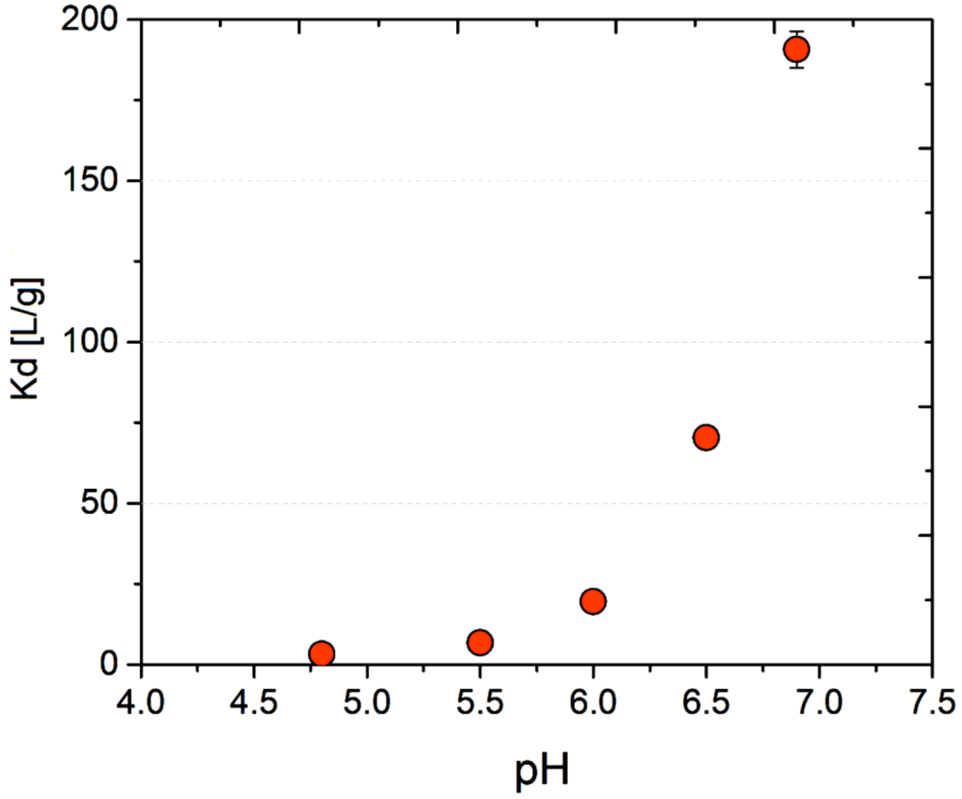

Figure S3. Preliminary batch equilibrium experiments using the ^{64}Cu-radiolabeled Cu(MCPA)$_2$ solution and the column filling material were performed to try to reproduce the batch equilibrium adsorption experiment results. The figure shows the distribution coefficient K_d vs. pH determined by the particle reactive [^{64}Cu]Cu(MCPA)$_2$ tracer batch-equilibrated for 24 h with 10 g column filling material (equal to 100 mg goethite) suspended in 25 mL of 10 M KF/1 mM NaNO$_3$ solution. The increase in K_d is due to polymerization of the Cu-MCPA complex (red curve in Figure 1 of main text).

The PET experiment. The ClearPET is a high-performance small animal PET scanner that has been developed by the Crystal Clear Collaboration (CCC) and was commercially available through the *Raytest* group (Sempere-Roldan et al., 2007). The ClearPET is characterized by the following performance data:

- 10240 double-layered miniaturized (2mm×2mm×10 mm) detector crystals, L(Y)SO/LuYAP:Ce in phoswich configuration providing DOI-Information
- Modular, 20 cassettes with each 4 PMTs (Hamamatsu R7600-M54)
- Rotating gantry (1×360° rotation per min = minimum frame length 60 s)
- Adjustable gantry diameter: (125 – 220) mm
- Field of View (FOV): adjustable diameter (70 – 144) mm, length 110 mm
- Activity range in FOV: ca. 100 kBq to 30 MBq (small) / 70 MBq (large)
- Detection threshold in the order of 3 kBq mL^{-1}
- Resolution CFOV 1.1 mm
- Max. sensitivity 4.5 %
- Open-Source software STIR, available via http://stir.sourceforge.net/

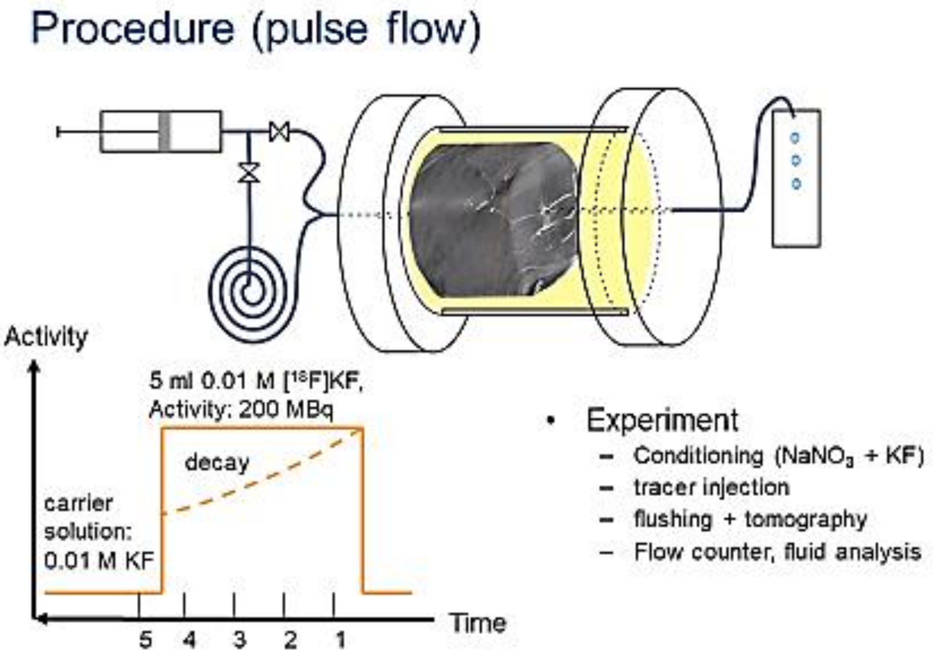

Figure S4. Principal workflow of the PET experiment

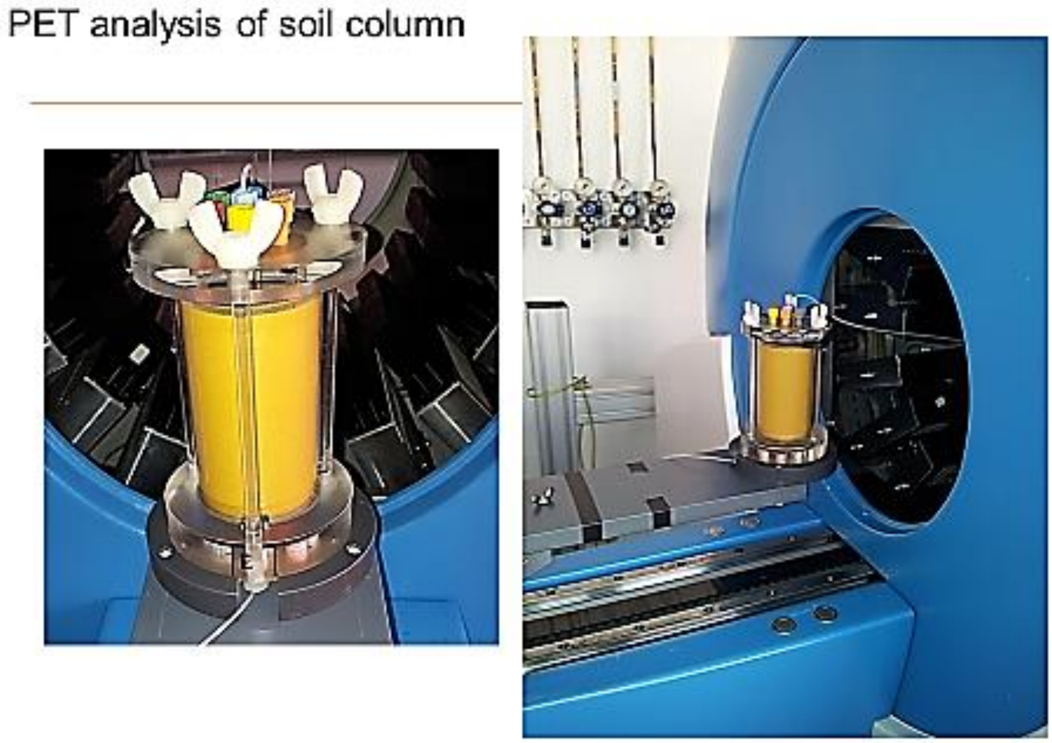

Figure S5. Photograph of the PET gantry with the soil column mounted vertically on the slide.

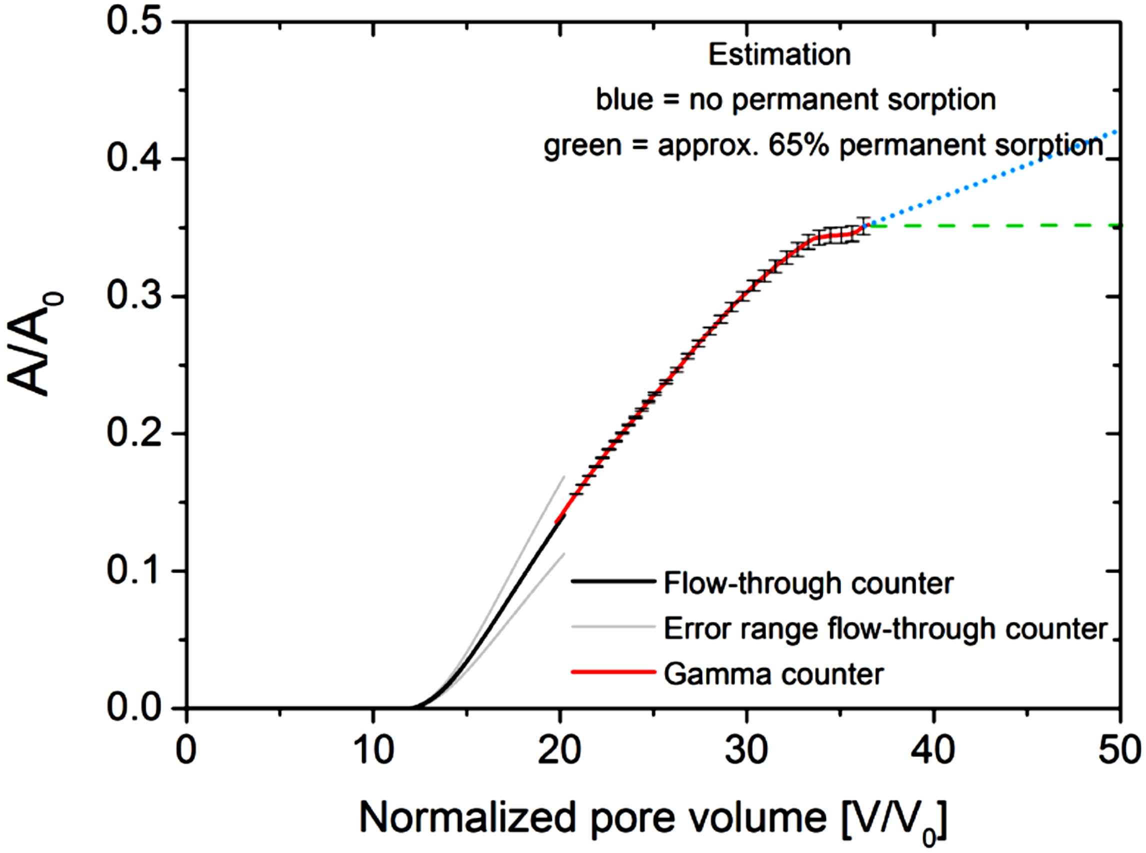

Figure S6. Global break-through curve for the ^{64}Cu tracer (decay-corrected) as measured at the outflow by a gamma-counter equipped with fraction sampler. The experiment #3 was terminated just after 50% of the tracer came out. The curve is extrapolated under assumption of 44% of tracer trapped (red curve) or no trapping (blue curve).

REFERENCES

Avila-Rodriguez, M.A., Nye, J.A. & Nickles, R.J. Simultaneous production of high specific activity ^{64}Cu and ^{61}Co with 11.4 MeV protons on enriched ^{64}Ni nuclei. *Appl. Radiat. Isotop*. **65**, 1115-1120, (2007).

Gustafsson, J.P. Visual MINTEQ 3.1, code available at http://vminteq.lwr.kth.se (2016).

Haberhauer, G., Pfeiffer, L. & Gerzabek, M.H. Influence of molecular structure on sorption of phenoxyalkanoic herbicides on soil and its particle size fractions. *J. Agric. Food Chem*. **48**, 3722-3727 (2000).

Haberhauer, G., Pfeiffer, L., Gerzabek, M.H., Kirchmann, H., Aquino, A.J.A., Tunega, D. & Lischka, H. Response of sorption processes of MCPA to the amount and origin of organic matter in a long-term field experiment. *Eur. J. Soil Sci*. **52**, 279-286 (2001)

Hiemstra, T. & Van Riemsdijk, W.H. On the relationship between charge distribution, surface hydration, and the structure of the interface of metal hydroxides. *J. Colloid Interf. Sci*. **301**, 1-18, (2006).

Iglesias, A., López, R., Gondar, D., Antelo, J., Fiol, S. & Arce, F. Effect of pH and ionic strength on the binding of paraquat and MCPA by soil fulvic and humic acids. *Chemosphere* **76**, 107-113 (2009)

Kersten M., Tunega D., Georgieva I., Vlasova N. & Branscheid R. Adsorption of the herbicide 4-chloro-2-methylphenoxyacetic acid (MCPA) by goethite. *Environ. Sci. Technol*. **48**, 11803-11810, (2014).

Klepka, M.T., Drzewiecka, A., Wolska, A. & Ferenc, W. XAS studies on Cu(II) complexes with derivatives of phenoxyacetic and benzoic acids. *Chem. Phys. Lett*. **553**, 59-63, (2012).

Lützenkirchen, J., Boily, J.F., Gunneriusson, L., Lövgren, L. & Sjöberg, S. Protonation of different goethite surfaces – Unified models for NaNO3 and NaCl media. *J. Colloid Interf. Sci*. **317**, 155-165, (2008).

Masbouh, F., Kabli, H., El Amine, M.E., El Jazouli, H., Ichou, Y.A. & Albourine, A. Potentiometric examination of complexation equilibria of some phenoxyacetic acid derivatives with Cu(II), Cd(II) and Zn(II). *Chem. Spec. Bioavail*. **18**, 117-122, (2006).

Paszko, T., Muszynski, P., Materska, M., Bojanowska, M., Kostecka, M. & Jackowska, I. Adsorption and degradation of phenoxyalkanoic acid herbicides in soils: a review. *Environ. Toxicol. Chem*. **35**, 271-286 (2016)

Sempere-Roldan, P., Chereul, E., Dietzel, O., Magnier, L., Pautrot, C., Rbah, L., Sappey-Marinier, D., Wagner, A., Rimmer, L., Janier, M., Tarazona, V. & Dietzel, G. Raytest ClearPET, a new generation small animal PET scanner. *Nucl. Instrum. Methods Phys. Res. A* **571**, 498-501, (2007).

Szelecsényi, F., Blessing, G. & Qaim, S.M., Excitation functions of proton induced nuclear reactions on enriched ^{61}Ni and ^{64}Ni: Possibility of production of no-carrier-added ^{61}Cu and ^{64}Cu at a small cyclotron. *Appl. Radiat. Isotop*. **44**, 575-580, (1993).

Thieme, S., Walther, M., Pietzsch, H.-J., Henninger, J., Preusche, S., Mäding, P. & Steinbach, J. Module-assisted preparation of ^{64}Cu with high specific activity. *Appl. Radiat. Isotop*. **70**, 602-608, (2012).

Weng, L., Van Riemsdijk, W.H. & Hiemstra, T. Cu^{2+} and Ca^{2+} adsorption to goethite in the presence of fulvic acids. *Geochim. Cosmochim. Acta* **72**, 5857-5870, (2008).
